# A Hybrid Butterfly Optimization Algorithm for Numerical Optimization Problems

**DOI:** 10.1155/2021/7981670

**Published:** 2021-12-24

**Authors:** Huan Zhou, Hao-Yu Cheng, Zheng-Lei Wei, Xin Zhao, An-Di Tang, Lei Xie

**Affiliations:** ^1^Aviation Engineering School, Air Force Engineering University, Xi'an, China; ^2^Unmanned System Research Institute, Northwestern Polytechnical University, Xi'an, China; ^3^China Aerodynamic Research and Development Center, Mianyang, China

## Abstract

The butterfly optimization algorithm (BOA) is a swarm-based metaheuristic algorithm inspired by the foraging behaviour and information sharing of butterflies. BOA has been applied to various fields of optimization problems due to its performance. However, BOA also suffers from drawbacks such as diminished population diversity and the tendency to get trapped in local optimum. In this paper, a hybrid butterfly optimization algorithm based on a Gaussian distribution estimation strategy, called GDEBOA, is proposed. A Gaussian distribution estimation strategy is used to sample dominant population information and thus modify the evolutionary direction of butterfly populations, improving the exploitation and exploration capabilities of the algorithm. To evaluate the superiority of the proposed algorithm, GDEBOA was compared with six state-of-the-art algorithms in CEC2017. In addition, GDEBOA was employed to solve the UAV path planning problem. The simulation results show that GDEBOA is highly competitive.

## 1. Introduction

Optimization problems exist in all aspects of our society, including business, engineering, and science. An optimization problem is the process of finding the best value of decision variables that satisfy the maximum or minimum objective value without violating the constraints. With the development of science and technology in these days, the optimization problems we encounter have become increasingly complex. These real-world optimization problems often involve many decision variables, complex nonlinear constraints and nonconvexity, dynamic objective functions, and expensive computational costs [1, 2]. Therefore, when we solve these problems using traditional gradient-based methods, we encounter many difficulties in achieving a satisfactory solution [3]. As the field of optimization has developed, metaheuristic algorithms have become increasingly popular. It has the property of achieving an optimal or near-optimal solution in a reasonable time and does not rely on problem-specific gradient information [4]. It is therefore widely used to solve various types of optimization problems, such as task planning [5–7], feature selection [8, 9], parameter optimization [10, 11], and image segmentation [12, 13].

In the last decades, many metaheuristic algorithms have been proposed and successfully applied to different domains. These algorithms can be divided into three categories: evolutionary-based algorithms, physical-based algorithms, and swarm-based algorithms. Evolution-based algorithms are a class of algorithms that simulate the laws of evolution in nature. The genetic algorithm (GA) [14] is a widely used evolutionary-based algorithm proposed by Holland. It updates populations by simulating the natural law of survival of the fittest. With the popularity of GA and GA variants, more and more evolutionary-based algorithms are continuously being proposed, including differential evolution (DE) [15], genetic programming (GP) [16], evolutionary strategies (ES) [17], and evolutionary programming (EP) [18]. Besides these evolutionary algorithms, some novel evolutionary-based algorithms have been proposed recently, such as artificial algae algorithm (AAA) [19], biogeography-based optimization (BBO) [20], and monkey king evolutionary (MKE) [21]. Physical-based algorithms simulate the laws of physics in nature or in the universe. Simulated annealing (SA) [22] inspired by annealing phenomena in metallurgy is one of the best-known physical-based algorithms. There are other physical-based algorithms proposed, including gravitational search algorithm (GSA) [23], nuclear reaction optimization (NRO) [24], water cycle algorithm (WCA) [25], and sine cosine algorithm (SCA) [26]. Swarm-based algorithms simulate social behaviour such as self-organization and division of labour in species. The particle swarm optimization (PSO) [27]and ant colony optimization (ACO) [28] are two classic swarm-based algorithms. Inspired by these two algorithms, more and more scholars are conducting research on this subject and proposing different swarm-based algorithms. The popularity of PSO and ACO has prompted more researchers to propose new swarm-based metaheuristics. Mirjalili et al. proposed grey wolf optimizer based on the collaborative foraging of grey wolves [29]. Wang et al. proposed monarch butterfly optimization inspired by the migratory activity of monarch butterflies [30]. Inspired by the spiral foraging and parabolic foraging of tuna, Xie et al. proposed the tuna swarm optimization [31]. In addition to the above three types of algorithms, a class of human-based metaheuristics is beginning to emerge. These algorithms are inspired by the characteristics of human activity. Teaching-learning-based optimization (TLBO) [32], inspired by traditional teaching methods, is a typical example of this category of metaheuristic algorithms. Other human-based metaheuristics include social evolution and learning optimization (SELO) [33], heap-based optimizers (HBO) [34], political optimizers (PO) [35], and many others. The butterfly optimization algorithm (BOA) is a swarm-based metaheuristic algorithm proposed by Arora and Singh [36]. BOA establishes an exploitation and exploration process based on the foraging behaviour and information-sharing strategies of butterflies. Although the BOA can perform exploitation and exploration operations, the basic BOA suffers from diminished population diversity and a tendency to fall into local optimum. Meanwhile, the No free lunch theory (NFL) [37] states that no single algorithm can solve all optimization problems perfectly. These factors encourage us to further enhance and improve the performance of BOA.

Metaheuristics have a common property: they find optimal solutions by exploiting and exploring the search space. Exploitation dominates and will weaken exploration. But when exploration is enhanced, exploitation is weakened. So, we need to improve algorithm performance by balancing exploitation and exploration. The improvements to the algorithm focus on three main areas. The first one is the optimization of the algorithm's parameter settings. Fan et al. [38] adjusted the fragrance factor of BOA and proposed an adaptive fragrance factor update method to enhance the convergence of BOA. Tang et al. [39] proposed the use of chaotic mapping operators to replace the alert value of the sparrow search algorithm for the purpose of balancing exploitation and exploration. Fan et al. [32] presented a new nonlinear step-factor control parameter strategy to further enhance the global search capability of the marine predator algorithm. The second is to use some techniques from other fields to improve performance. The fractional order is an effective tool that has been used in other areas [33, 40]. Yousri et al. [34] proposed an enhanced Harris hawk optimization based on fractional-order calculus memory. Elaziz et al. [35] improved the initial population of the Harris hawk optimizer using fractional-order Gaussians and 2 × mod1 chaotic mappings. The third is to use other operators to improve the original algorithm. Wang et al. [41] proposed a hybrid metaheuristic algorithm for butterfly and flower pollination based on a reciprocal mechanism. Houssein et al. [42] proposed a variant of the slime mould algorithm with hybrid adaptive guided differential evolution in order to overcome the disadvantages of unbalanced exploitation and exploration. Inspired by these hybrid variants, this paper proposes a BOA variant with hybrid distribution estimation strategy, GDEBOA. GDEBOA uses a Gaussian probability model to describe the distribution of dominant populations and to guide the direction of evolution, improving the performance of the basic BOA. The performance of GDEBOA was evaluated on the CEC2017 test suite and compared with seven state-of-the-art algorithms. The superiority of the proposed algorithm is verified by numerical analysis, convergence analysis, stability analysis, and statistical analysis. In addition, GDEBOA is applied to the UAV route planning problem to further validate the algorithm's ability to solve real-world optimization problems.

The remainder of this paper is organized as follows. A review of the basic BOA is presented in Section 2.  Section 3 provides a detailed description of the proposed GDEBOA. In Section 4, the effectiveness of the proposed improvement strategy is verified using CEC 2017 test suite. Furthermore, GDEBOA is applied to solve the UAV route planning problem in Section 5. Finally, we summarize this work in Section 6 and offer directions for future research.

## 2. Butterfly Optimization Algorithm

The butterfly optimization algorithm is a swarm-based metaheuristic algorithm proposed by Arora et al. The algorithm builds a model of butterfly foraging and mating. BOA proposes three hypotheses: (1) all butterflies emit fragrance and are attracted to each other; (2) each butterfly moves randomly or towards the butterfly with the most scent; and (3) the stimulus intensity of the butterfly is determined by the landscape of the fitness function. As the butterflies move, the fragrance changes with them. All butterflies form a fragrance network, and when they do not feel the fragrance network, they fly randomly, which is called the global search phase. As the butterflies approach the butterfly with the greatest concentration of fragrance, this phase is called the local search phase. BOA solves the optimization problem through global and local search with the following mathematical model.

The fragrance of the butterfly is expressed as a function of the physical intensity of the stimulus, described as follows:

(1) $$\begin{array}{c} f_{i}=cI^{a},\;i=1,2,\ldots ,NP. \end{array}$$

where *f*_*i*_ represents the butterfly fragrance, *c* represents the sensory modality, *I* represents the stimulus intensity, *a* is a power exponent with a value from 0 to 1, and *NP* denotes the number of butterflies. The mathematical model of the global and local search phases of BOA is represented as follows:

(2) $$\begin{array}{c} X_{i}^{t+1}=\;X_{i}^{t}\;+\;\left(r^{2}\;\times \;X_{\text{best}}^{t}\;-\;X_{i}^{t}\;\right)\;\times f_{i}, \end{array}$$

(3) $$\begin{array}{c} X_{i}^{t+1}=\;X_{i}^{t}\;+\;\left(r^{2}\;\times \;X_{j}^{t}\;-\;X_{k}^{t}\;\right)\;\times f_{i}, \end{array}$$

where *X*_*i*_^*t*^ denotes the position of the *i*^th^ butterfly in the *t*^th^ iteration, *X*_best_^*t*^ denotes the global optimal individual, *r* ∈ (0,1) is a random number, and *X*_*j*_^*t*^ and *X*_*k*_^*t*^ are the *j*^th^ individual and the *k*^th^ individual selected randomly. BOA constantly executes two search strategies during the search process. Therefore, a switching probability *p* is introduced to control the switching of the two strategies. The pseudocode for BOA is given in Algorithm 1.

## 3. Proposed GDEBOA

To overcome the shortcomings of the basic butterfly optimization algorithm, a modified butterfly optimization algorithm, called GDEBOA, is proposed in this paper. The combination of the Gaussian distribution estimation algorithm and BOA provides a solution to the problem of unbalanced exploitation and exploration capabilities that exist in BOA. Here, this paper employs a Gaussian distribution estimation strategy as an alternative to the global search strategy in BOA. The GDE is used to sample the dominant population and guide the evolutionary direction of the algorithm while increasing population diversity. The improved strategies and GDEBOA are described in detail below.

### 3.1. Gaussian Distribution Estimation

**Figure 1:**
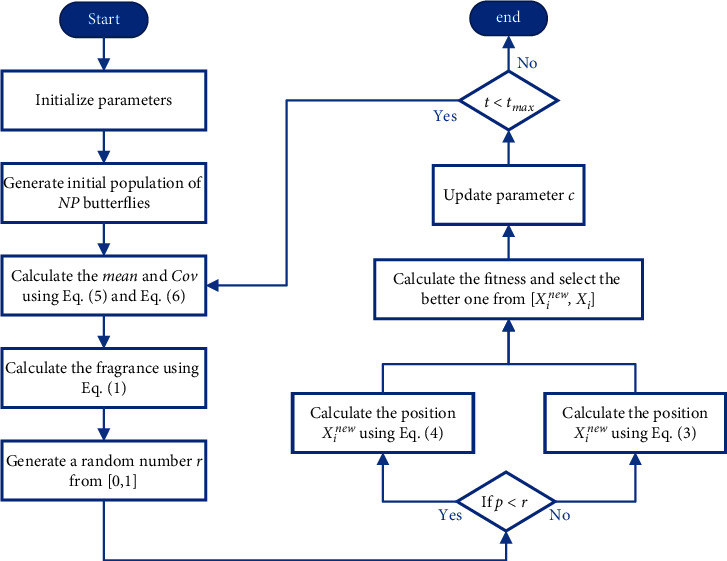
Flowchart of GEDBOA.

The Gaussian distribution estimation strategy represents inter-individual relationships through a probabilistic model. The strategy uses the current dominant population to calculate the probability distribution model and generates new offspring populations based on the probability distribution model sampling, eventually obtaining the optimal solution by continuous iteration. In this paper, the distribution model is estimated using a weighted maximum likelihood estimation method, and the top one-half population that performs better is taken as the dominant population. The mathematical model of this strategy is described as follows:

(4) $$\begin{array}{c} X_{i}^{t+1}=mean+y,\;y∼N\left(0,Cov\right), \end{array}$$

(5) $$\begin{array}{c} mean=\frac{\left(X_{\text{best}}+X_{\text{mean}}^{t}+X_{i}^{t}\right)}{3}, \end{array}$$

(6) $$\begin{array}{c} Cov\left(i\right)=\frac{1}{NP/2}\sum_{i=1}^{NP/2}\left(X_{i}^{t+1}-X_{\text{mean}}^{t}\right)\times {\left(X_{i}^{t}-X_{\text{mean}}^{t}\right)}^{T}, \end{array}$$

(7) $$\begin{array}{c} X_{\text{mean}}^{t}=\sum_{i=1}^{NP/2}\omega_{i}\times X_{i}^{t}, \end{array}$$

(8) $$\begin{array}{c} \omega_{i}=\frac{\ln\left(0.5\text{NP}+0.5\right)-\ln\left(i\right)}{\sum_{i=1}^{NP/2}\left(\ln\left(0.5\text{NP}+0.5\right)-\ln\left(i\right)\right)}, \end{array}$$

where *X*_mean_^*t*^ denotes the weighted position of the dominant population and *ω* denotes the weight coefficient in the dominant population in descending order of fitness values. **Cov** is the weighted covariance matrix of the dominant populations. The pseudocode and flowchart of the proposed GDEBOA are shown in Algorithm 2 and Figure 1.

## 4. Numerical Experiment and Analysis

In order to fully validate the superior performance of the proposed GDEBOA, the algorithm was tested using the IEEE CEC2017 single-objective test function. The CEC2017 test suite consists of 28 test functions. F1 is a unimodal function with only one global optimum solution and is used to verify the local search capability of the algorithm. F2–F8 are multimodal functions and are primarily used to test the ability of an algorithm to get outside of a local optimum. F9–F17 and F18–F28 are hybrid and composite functions, respectively, and can be used to test the potential of an algorithm to solve complex optimization problems in the real world. The definition of functions and optima is given in Table 1.

Seven state-of-the-art metaheuristics were used for comparison with GDEBOA, including artificial ecosystem-based optimization (AEO) [43], grey wolf optimizer (GWO) [29], Harris hawks optimization (HHO) [44], arithmetic optimization algorithm (AOA) [45], slime mould algorithm (SMA) [46], Manta ray foraging optimization (MRFO) [47], and pathfinder algorithm (PFA) [48]. In the CEC2017 test, the maximum number of iterations is 600, and the population size is 500. All the algorithm parameters were set to be the same as in the original literature, as shown in Table 2. All algorithms were run 51 times independently, and the experimental results were recorded as shown in Table 3. In this paper, the performance of GDEBOA was evaluated comprehensively by numerical analysis, convergence analysis, stability analysis, Wilcoxon test, and Friedman test. The experiments in this paper were conducted on a computer with an AMD R7 4800U processor and 16 GB RAM. Programming was performed using MATLAB R2016b.

The results in Table 3 show that GDEBOA performs best on the unimodal function F1. Although GDEBOA does not consistently achieve the optimal solution, it provides the best solution among the nine algorithms. Compared to BOA, the performance of GDEBOA is significantly improved, indicating that the improvement strategy is effective in enhancing the exploitation capability. In the multimodal functions F2–F8, each algorithm performs differently. GDEBOA performs best on F2, F4, and F7. GWO provides the best solution on F3, F6, and F8. SMA achieves the optimal solution on F5. Significantly, GDEBOA outperforms BOA in all multimodal functions, which indicates that GDEBOA has a strong global search capability. The improved strategy can effectively enhance BOA's ability to explore the solution space. The GDEBOA performs best in most hybrid and composite functions. Specifically, GDEBOA achieved satisfactory results in 7 of the 10 hybrid functions. The GDEBOA achieved optimal solutions on 5 of the 10 composite functions. Compared to BOA, GDEBOA only performed worse on F19. The analysis of the results for the hybrid and composite functions shows that GDEBOA has a good balance of exploitation and exploration capabilities and is able to solve complex optimization problems effectively.

**Figure 2:**
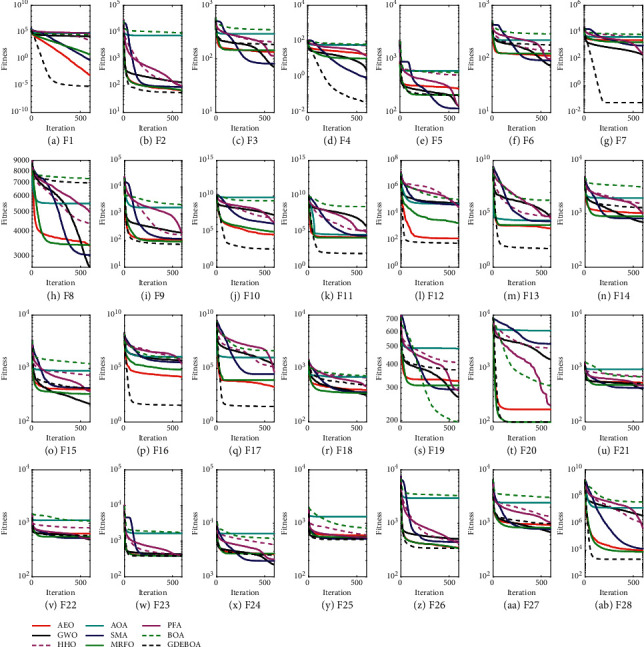
Convergence curves of the GDEBOA and other algorithms for CEC2017.

Convergence speed and convergence accuracy are important indicators of an algorithm's performance. Convergence analysis provides information on how the algorithm has changed over a process of iterations.  Figure 2 shows the average fitness convergence curves for F1–F28 based on the results of all algorithms solving the test suite 51 times. The results in Figure 2 show that GDEBOA has a faster convergence speed and better convergence accuracy compared to other algorithms. Specifically, GDEBOA outperformed all comparison algorithms in terms of convergence accuracy and convergence speed on 16 functions. Notably, although GDEBOA did not perform best on all functions, GDEBOA outperformed BOA on the 27 out of 28 functions, indicating that the improvement strategy proposed in this paper better balances exploitation and exploration.

**Figure 3:**
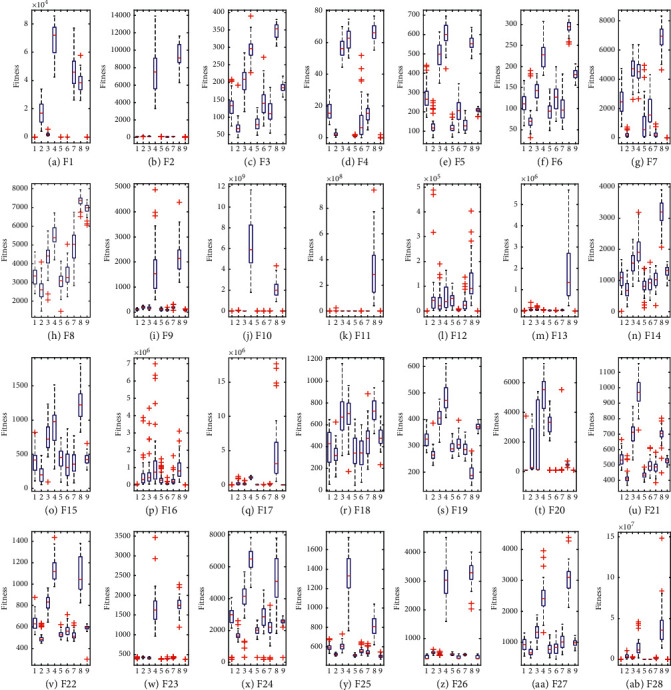
Boxplots analysis for CEC2017.

In addition, in order to analyse the distribution characteristics of GDEBOA's solutions when solving the functions, box diagrams are shown in Figure 3 based on the results of the nine algorithms solving the CEC2017 test set 51 times independently. For most of the test functions, the minimum, maximum, and median values obtained by GDEBOA are the same as the optimal solutions, which indicates that the solutions obtained by GDEBOA are more centrally distributed and more stable. Compared to BOA, GDEBOA solves the function with fewer bad values and a more concentrated distribution of solutions, indicating that GDEBOA has achieved a balance between exploitation and exploration.

Although the superiority of GDEBOA was demonstrated by comparisons of the mean and standard deviation, the literature [49, 50] demonstrates that these comparisons are not adequate. To further verify the differences between GDEBOA and other algorithms, the Wilcoxon signed-rank test was employed.  Table 4 shows the results of the Wilcoxon signed-rank test with significance level *P*=0.05. The term *R*+ indicates the extent to which GDEBOA outperforms the comparison algorithm, while *R*− indicates the opposite. The symbol “+/=/−” indicates that GDEBOA performs better than, similar to, and worse than the comparison algorithm.

From Table 4, GDEBOA outperforms the comparison algorithm on most functions. Numerically, GDEBOA is superior to MRFO on 14 functions. GDEBOA outperforms GWO and PFA on 16 functions. Compared to HHO and AOA, GDEBOA performs better on 27 and 28 functions. In particular, GDEBOA offers better solutions than BOA on all functions, except F9. In general, GDEBOA shows a superior performance.

## 5. UAV Route Planning

In this section, the application of GDEBOA to UAV route planning is discussed in detail. The UAV route planning problem aims to minimize the cost of carrying out the mission, which can be considered as a multi-constraint optimization problem. The route planning model is described in detail in the following section.

### 5.1. Cost Function

Considering the actual UAV flight scenario, we think that the UAV should reach the target as fast as possible while being free from threats during the flight. The cost function is described as the total of two functions and two constraints.

(9) $$\begin{array}{c} \min J=\omega_{1}\cdot {\text{Cost}}_{p}+\omega_{2}\cdot {\text{Cost}}_{h}+\eta \cdot \text{Penalty},\\ \text{Penalty}=\sum_{i=1}^{n}c_{i},\;n=1,2,\\ c_{i}=\left\{\begin{array}{cc} 0, & \text{Satisfing constraints,}\\ 1, & \text{No satisfing constraints,} \end{array}\right) \end{array}$$

where *J* is the total cost function, Cost_*p*_ denotes the flight distance cost, Cost_*h*_ denotes the height cost, *ω*_1_ and *ω*_2_ are the weighting factors of two costs that satisfy *ω*_1_+*ω*_2_=1, Penalty denotes the number of constraint violations, and *η* is the penalty function factor for converting the constrained optimization problem into an unconstrained optimization problem.

The faster the UAV reaches the target, the better it is for the mission, so the path cost is represented by the sum of the route segments.

(10) $$\begin{array}{c} {\text{Cost}}_{p}=\sum_{i=1}^{NP-1}{\text{Dis}}_{i}, \end{array}$$

where Dis_*i*_ is the length of the *i*^th^ route segment.

Besides, it is not beneficial for UAV to fly too high to avoid the threat. Therefore, the UAV needs to maintain low height flight. The corresponding height cost function is described as follows:

(11) $$\begin{array}{c} {\text{Cost}}_{h}=\sum_{i=1}^{NP}z_{i}, \end{array}$$

where *z*_*i*_ is the height corresponding to the *i*^th^ route point.

Due to the limitation of UAV's maneuverability, the turn angle and climb angle of the actual flight should be less than the theoretical maximum.

(12) $$\begin{array}{c} {\text{Constraint}}_{1}=\max\left|\varphi_{ij}\right|-\varphi_{\max}\leq 0,\\ {\text{Constraint}}_{2}=\max\left|\theta_{ij}\right|-\theta_{\max}\leq 0, \end{array}$$

where *φ*_*ij*_ and *θ*_*ij*_ are the turn angle and climb angle between each track segment, respectively, and *φ*_max_ and *θ*_max_ are the corresponding theoretical maximum values.

### 5.2. Simulation Experiment

**Figure 4:**
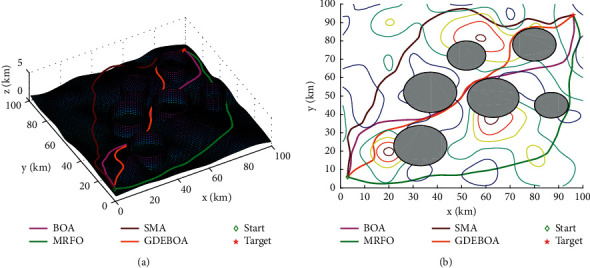
The best path of each algorithm: (a) path in three-dimensional space; (b) path in two-dimensional space.

In this subsection, we will solve the route planning model using GDEBOA and compare the results with BOA, MRFO, and SMA. All the programs were written using MATLAB R2016b and run on a Windows 10 platform with AMD R7 4700U 16 GB. For fair comparison, the parameters of each algorithm are set according to the original literature. The maximum iteration is 300, the number of populations is 50, and each algorithm is executed 30 times independently. The location of the threat is shown in Table 5. The best paths generated by MRFO, HHO, BOA, and GDEBOA are shown in Figure 4. The best values, mean, standard deviation, and success rate for 30 independent runs are shown in Table 6.

**Figure 5:**
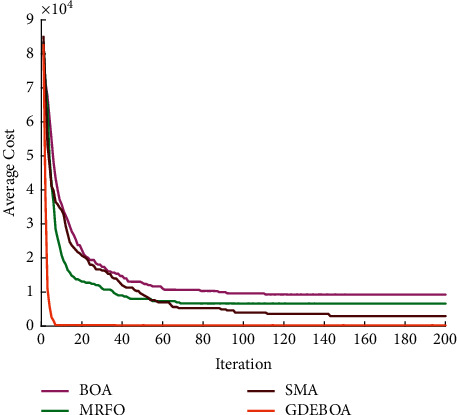
Convergence curves for four algorithms.

It is clear from analyzing the test results that all algorithms can give a safe flight route. This shows that the route planning model proposed in this paper is feasible. The path found by these algorithms is different as can be seen in Figure 4. Obviously, GDEBOA can find a safe path at lower cost. Also, GDEBOA can consistently find safe flight paths in 30 tests, while there are failures in the other algorithms. This indicates that GDEBOA is superior to the other three algorithms. The average cost convergence curve is plotted according to the statistical results, as shown in Figure 5. GDEBOA has faster convergence speed and better convergence accuracy.

## 6. Conclusions

In this paper, we propose a variant of BOA using a distribution estimation strategy, called GDEBOA, to solve the global optimization problem. The performance of BOA is enhanced by using the distribution estimation strategy to sample the evolutionary information of the dominant population and to guide the direction of individual evolution. To evaluate the effectiveness of the improved strategy and the superiority of GDEBOA, it was validated using the CEC2017 test suite. It was also compared with seven advanced algorithms through numerical analysis, convergence analysis, stability analysis, and statistical tests. The simulation results show that GDEBOA balances exploitation and exploration and is competitive with other algorithms. In addition, GDEBOA is applied to solve the UAV route planning problem. Simulation results show that GDEBOA can stably find paths with better quality, demonstrating the ability of GDEBOA to solve real-world optimization problems. Of course, there is still potential for enhancement of GDEBOA. The calculation of the covariance matrix leads to an increase in the computational cost of the algorithm. Therefore, how to reduce the cost of computing while maintaining performance is something we need to investigate further.

In future work, we plan to further apply GDEBOA to cooperative multi-UAV route planning and real-time route planning. Moreover, we plan to develop the multi-objective and binary versions of GDEBOA to solve optimization problems in other fields.

## Figures and Tables

**Algorithm 1:**
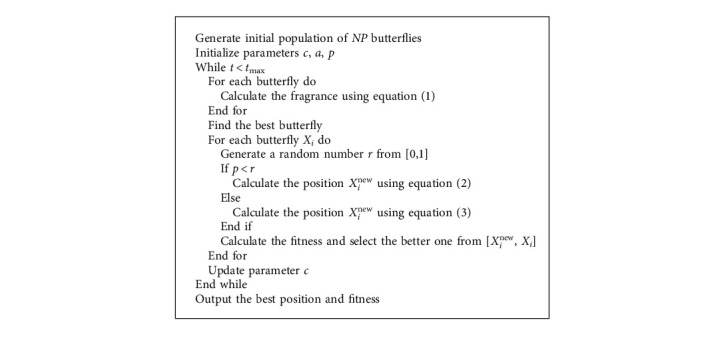
Butterfly optimization algorithm.

**Algorithm 2:**
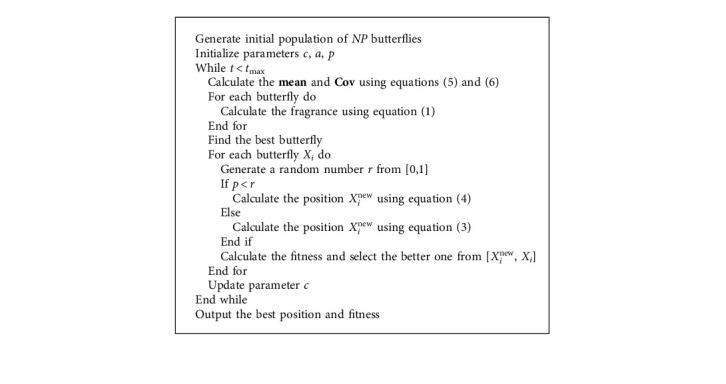
GDEBOA.

**Table 1:** Descriptions of CEC 2017 test suite.

Type	No.	Description	Fi^*∗*^
Unimodal functions	1	Shifted and rotated bent Cigar function	300
Unimodal functions	2	Shifted and rotated Rosenbrock's function	400
	3	Shifted and rotated Rastrigin's function	500
	4	Shifted and rotated expanded Scaffer's F6 function	600
	5	Shifted and rotated Lunacek bi-Rastrigin function	700
	6	Shifted and rotated noncontinuous Rastrigin's function	800
	7	Shifted and rotated Levy function	900
	8	Shifted and rotated Schwefel's function	1000

Hybrid functions	9	Hybrid function 1 (*N* = 3)	1100
	10	Hybrid function 2 (*N* = 3)	1200
	11	Hybrid function 3 (*N* = 3)	1300
	12	Hybrid function 4 (*N* = 4)	1400
	13	Hybrid function 5 (*N* = 4)	1500
	14	Hybrid function 6 (*N* = 4)	1600
	15	Hybrid function 6 (*N* = 5)	1700
	16	Hybrid function 6 (*N* = 5)	1800
	17	Hybrid function 6 (*N* = 5)	1900
	18	Hybrid function 6 (*N* = 6)	2000

Composite functions	19	Composition function 1 (*N* = 3)	2100
	20	Composition function 2 (*N* = 3)	2200
	21	Composition function 3 (*N* = 4)	2300
	22	Composition function 4 (*N* = 4)	2400
	23	Composition function 5 (*N* = 5)	2500
	24	Composition function 6 (*N* = 5)	2600
	25	Composition function 7 (*N* = 6)	2700
	26	Composition function 8 (*N* = 6)	2800
	27	Composition function 9 (*N* = 3)	2900
	28	Composition function 10 (*N* = 3)	3000

**Table 2:** Algorithms used for comparative analysis and their parameter settings.

Algorithm	Parameters
AEO	No parameters
GWO	*a*=2 (linearly decreased over iterations)
HHO	*β*=1.5, *E*_0_ ∈ [−1,1]
AOA	Mop_max_=1, Mop_min_=0.2, *C*=1, *α*=5, Mu=0.499
SMA	*z*=0.03
MRFO	*S*=2
PFA	*u*_1_=−1+2rand, *u*_2_=−1+2rand

**Table 3:** Statistical results of the nine comparison algorithms for CEC2017.

No.	Index	AEO	GWO	HHO	AOA	SMA	MRFO	PFA	BOA	GDEBOA
F1	Mean	9.01*E* − 04	1.74*E* + 04	1.68*E* + 03	6.91*E* + 04	4.94*E* − 01	6.61*E* + 00	4.66*E* + 04	3.82 *E* +04	7.70*E* − 06
	Std.	1.89*E* − 03	7.95*E* + 03	7.95*E* + 02	1.15*E* + 04	3.18*E* − 01	4.23*E* + 00	1.22*E* + 04	6.97 *E* + 03	9.81*E* − 07
	Rank	2	6	5	9	3	4	8	7	1
F2	Mean	6.65*E* + 01	1.35*E* + 02	1.23*E* + 02	7.61*E* + 03	8.99*E* + 01	6.70*E* + 01	9.80*E* + 01	9.33*E* + 03	5.49*E* + 01
	Std.	3.72*E* + 01	2.53*E* + 01	3.33*E* + 01	2.45*E* + 03	5.12*E* + 00	4.09*E* + 01	1.77*E* + 01	1.29*E* + 03	2.08*E* + 01
	Rank	2	7	6	8	4	3	5	9	1
F3	Mean	1.34*E* + 02	7.12*E* + 01	2.05*E* + 02	2.95*E* + 02	8.17*E* + 01	1.46*E* + 02	1.14*E* + 02	3.49*E* + 02	1.86*E* + 02
	Std.	3.17*E* + 01	2.25*E* + 01	3.62*E* + 01	3.20*E* + 01	1.99*E* + 01	4.09*E* + 01	3.11*E* + 01	2.16*E* + 01	1.18*E* + 01
	Rank	4	1	7	8	2	5	3	9	6
F4	Mean	1.67*E* + 01	2.28*E* + 00	5.62*E* + 01	6.21*E* + 01	7.35*E* − 01	1.05*E* + 01	1.47*E* + 01	6.63*E* + 01	4.03*E* − 02
	Std.	5.63*E* + 00	1.21*E* + 00	5.92*E* + 00	6.71*E* + 00	3.21*E* − 01	1.21*E* + 01	4.99*E* + 00	5.76*E* + 00	2.51*E* − 01
	Rank	6	3	7	8	2	4	5	9	1
F5	Mean	2.75*E* + 02	1.31*E* + 02	4.98*E* + 02	6.00*E* + 02	1.18*E* + 02	2.12*E* + 02	1.34*E* + 02	5.57*E* + 02	2.09*E* + 02
	Std.	6.77*E* + 01	4.45*E* + 01	6.57*E* + 01	5.66*E* + 01	2.40*E* + 01	6.09*E* + 01	3.12*E* + 01	3.17*E* + 01	1.14*E* + 01
	Rank	6	2	7	9	1	5	3	8	4
F6	Mean	1.13*E* + 02	7.52*E* + 01	1.40*E* + 02	2.25*E* + 02	9.39*E* + 01	1.27*E* + 02	9.97*E* + 01	2.93*E* + 02	1.83*E* + 02
	Std.	2.33*E* + 01	3.07*E* + 01	2.13*E* + 01	2.67*E* + 01	2.03*E* + 01	3.29*E* + 01	2.66*E* + 01	1.54*E* + 01	1.11*E* + 01
	Rank	4	1	6	8	2	5	3	9	7
F7	Mean	2.49*E* + 03	2.05*E* + 02	4.69*E* + 03	4.50*E* + 03	9.95*E* + 02	1.77*E* + 03	2.28*E* + 02	6.82*E* + 03	5.35*E* − 02
	Std.	9.16*E* + 02	1.62*E* + 02	8.28*E* + 02	7.24*E* + 02	1.22*E* + 03	1.02*E* + 03	1.83*E* + 02	8.69*E* + 02	1.74*E* − 01
	Rank	6	2	8	7	4	5	3	9	1
F8	Mean	3.37*E* + 03	2.64*E* + 03	4.35*E* + 03	5.51*E* + 03	3.04*E* + 03	3.41*E* + 03	4.98*E* + 03	7.33*E* + 03	6.94*E* + 03
	Std.	5.42*E* + 02	5.39*E* + 02	7.25*E* + 02	5.83*E* + 02	5.06*E* + 02	6.00*E* + 02	9.01*E* + 02	2.85*E* + 02	2.87*E* + 02
	Rank	3	1	5	7	2	4	6	9	8
F9	Mean	1.09*E* + 02	1.96*E* + 02	1.61*E* + 02	1.72*E* + 03	1.16*E* + 02	9.20*E* + 01	1.91*E* + 02	2.19*E* + 03	7.09*E* + 01
	Std.	3.10*E* + 01	4.04*E* + 01	4.86*E* + 01	9.74*E* + 02	4.33*E* + 01	3.46*E* + 01	5.28*E* + 01	6.72*E* + 02	2.60*E* + 01
	Rank	3	7	5	8	4	2	6	9	1
F10	Mean	3.74*E* + 04	2.18*E* + 07	7.61*E* + 06	6.27*E* + 09	1.31*E* + 06	9.00*E* + 04	1.88*E* + 06	2.08*E* + 09	3.49*E* + 02
	Std.	3.35*E* + 04	1.87*E* + 07	4.21*E* + 06	2.56*E* + 09	1.09*E* + 06	7.44*E* + 04	1.97*E* + 06	7.43*E* + 08	2.52*E* + 02
	Rank	2	7	6	9	4	3	5	8	1
F11	Mean	1.51*E* + 04	5.50*E* + 05	1.51*E* + 05	3.80*E* + 04	2.71*E* + 04	1.36*E* + 04	7.54*E* + 04	3.15*E* + 08	8.30*E* + 01
	Std.	1.46*E* + 04	3.30*E* + 06	9.05*E* + 04	1.71*E* + 04	2.64*E* + 04	1.62*E* + 04	4.12*E* + 04	2.10*E* + 08	2.77*E* + 01
	Rank	3	8	7	5	4	2	6	9	1
F12	Mean	1.40*E* + 02	6.15*E* + 04	3.82*E* + 04	5.72*E* + 04	4.71*E* + 04	2.01*E* + 03	3.00*E* + 04	1.19*E* + 05	6.29*E* + 01
	Std.	4.36*E* + 01	9.79*E* + 04	4.25*E* + 04	4.92*E* + 04	2.84*E* + 04	2.19*E* + 03	2.94*E* + 04	7.62*E* + 04	7.46*E* + 00
	Rank	2	8	5	7	6	3	4	9	1
F13	Mean	4.10*E* + 03	5.46*E* + 04	6.86*E* + 04	2.35*E* + 04	1.99*E* + 04	8.64*E* + 03	3.35*E* + 04	1.82*E* + 06	5.54*E* + 01
	Std.	4.49*E* + 03	5.70*E* + 04	4.86*E* + 04	1.22*E* + 04	1.57*E* + 04	9.15*E* + 03	1.77*E* + 04	1.46*E* + 06	1.94*E* + 01
	Rank	2	7	8	5	4	3	6	9	1
F14	Mean	1.03*E* + 03	6.98*E* + 02	1.55*E* + 03	1.98*E* + 03	8.18*E* + 02	8.93*E* + 02	1.00*E* + 03	3.18*E* + 03	1.30*E* + 03
	Std.	3.11*E* + 02	2.59*E* + 02	3.56*E* + 02	5.09*E* + 02	2.83*E* + 02	2.63*E* + 02	2.63*E* + 02	4.12*E* + 02	1.80*E* + 02
	Rank	5	1	7	8	2	3	4	9	6
F15	Mean	4.06*E* + 02	2.17*E* + 02	7.48*E* + 02	9.12*E* + 02	4.34*E* + 02	3.36*E* + 02	3.77*E* + 02	1.22*E* + 03	4.31*E* + 02
	Std.	1.84*E* + 02	1.23*E* + 02	2.19*E* + 02	2.67*E* + 02	1.64*E* + 02	2.02*E* + 02	1.71*E* + 02	2.49*E* + 02	7.95*E* + 01
	Rank	4	1	7	8	6	2	3	9	5
F16	Mean	1.70*E* + 04	5.57*E* + 05	6.90*E* + 05	1.29*E* + 06	3.75*E* + 05	7.66*E* + 04	2.75*E* + 05	9.60*E* + 05	3.68*E* + 01
	Std.	1.28*E* + 04	7.70*E* + 05	8.77*E* + 05	1.60*E* + 06	3.55*E* + 05	6.45*E* + 04	2.82*E* + 05	6.22*E* + 05	4.62*E* + 00
	Rank	2	6	7	9	5	3	4	8	1
F17	Mean	1.94*E* + 03	2.42*E* + 05	1.46*E* + 05	1.08*E* + 06	3.00*E* + 04	8.42*E* + 03	4.45*E* + 04	4.61*E* + 06	2.79*E* + 01
	Std.	3.07*E* + 03	2.98*E* + 05	1.42*E* + 05	1.39*E* + 05	2.11*E* + 04	1.07*E* + 04	3.91*E* + 04	4.06*E* + 06	2.57*E* + 00
	Rank	2	7	6	8	4	3	5	9	1
F18	Mean	3.95*E* + 02	3.20*E* + 02	6.71*E* + 02	6.94*E* + 02	3.59*E* + 02	3.45*E* + 02	4.61*E* + 02	7.29*E* + 02	4.90*E* + 02
	Std.	1.62*E* + 02	1.03*E* + 02	2.01*E* + 02	1.54*E* + 02	1.59*E* + 02	1.33*E* + 02	1.52*E* + 02	9.88*E* + 01	9.82*E* + 01
	Rank	4	1	7	8	3	2	5	9	6
F19	Mean	3.26*E* + 02	2.67*E* + 02	4.06*E* + 02	4.87*E* + 02	2.93*E* + 02	3.09*E* + 02	2.90*E* + 02	1.97*E* + 02	3.73*E* + 02
	Std.	3.26*E* + 01	2.45*E* + 01	3.51*E* + 01	5.23*E* + 01	2.17*E* + 01	2.95*E* + 01	2.62*E* + 01	3.01*E* + 01	1.21*E* + 01
	Rank	6	2	8	9	4	5	3	1	7
F20	Mean	1.73*E* + 02	1.49*E* + 03	2.39*E* + 03	5.13*E* + 03	2.90*E* + 03	1.00*E* + 02	2.08*E* + 02	4.71*E* + 02	1.00*E* + 02
	Std.	5.11*E* + 02	1.42*E* + 03	2.37*E* + 03	1.21*E* + 03	1.36*E* + 03	7.40*E* − 01	7.61*E* + 02	7.76*E* + 01	2.31*E* − 04
	Rank	3	6	7	9	8	2	4	5	1
F21	Mean	5.41*E* + 02	4.18*E* + 02	7.05*E* + 02	9.68*E* + 02	4.35*E* + 02	4.95*E* + 02	4.85*E* + 02	6.97*E* + 02	5.28*E* + 02
	Std.	4.86*E* + 01	3.60*E* + 01	7.35*E* + 01	9.10*E* + 01	1.95*E* + 01	4.15*E* + 01	4.09*E* + 01	5.59*E* + 01	1.59*E* + 01
	Rank	6	1	8	9	2	4	3	7	5
F22	Mean	6.41*E* + 02	5.02*E* + 02	8.26*E* + 02	1.14*E* + 03	5.30*E* + 02	5.65*E* + 02	5.24*E* + 02	1.10*E* + 03	5.89*E* + 02
	Std.	7.63*E* + 01	4.93*E* + 01	7.42*E* + 01	1.09*E* + 02	2.95*E* + 01	5.19*E* + 01	3.72*E* + 01	1.68*E* + 02	4.38*E* + 01
	Rank	6	1	7	9	3	4	2	8	5
F23	Mean	3.94*E* + 02	4.29*E* + 02	4.11*E* + 02	1.67*E* + 03	3.88*E* + 02	3.89*E* + 02	3.97*E* + 02	1.75*E* + 03	3.87*E* + 02
	Std.	1.47*E* + 01	1.79*E* + 01	1.87*E* + 01	4.55*E* + 02	1.69*E* + 00	8.17*E* + 00	1.72*E* + 01	2.01*E* + 02	6.50*E* − 01
	Rank	4	7	6	8	2	3	5	9	1
F24	Mean	2.62*E* + 03	1.69*E* + 03	3.94*E* + 03	6.40*E* + 03	1.98*E* + 03	2.71*E* + 03	2.12*E* + 03	5.21*E* + 03	2.54*E* + 03
	Std.	1.10*E* + 03	2.41*E* + 02	1.10*E* + 03	7.22*E* + 02	3.42*E* + 02	1.05*E* + 03	7.10*E* + 02	1.49*E* + 03	3.47*E* + 02
	Rank	5	1	7	9	2	6	3	8	4
F25	Mean	5.92*E* + 02	5.22*E* + 02	6.05*E* + 02	1.34*E* + 03	5.11*E* + 02	5.56*E* + 02	5.46*E* + 02	8.14*E* + 02	5.01*E* + 02
	Std.	3.05*E* + 01	9.78*E* + 00	4.00*E* + 01	2.14*E* + 02	1.17*E* + 01	2.78*E* + 01	2.85*E* + 01	9.81*E* + 01	1.52*E* + 01
	Rank	6	3	7	9	2	5	4	8	1
F26	Mean	3.49*E* + 02	5.11*E* + 02	4.62*E* + 02	2.95*E* + 03	4.46*E* + 02	3.42*E* + 02	4.31*E* + 02	3.28*E* + 03	3.39*E* + 02
	Std.	5.89*E* + 01	4.00*E* + 01	2.60*E* + 01	6.15*E* + 02	2.89*E* + 01	5.27*E* + 01	1.90*E* + 01	3.99*E* + 02	5.28*E* + 01
	Rank	3	7	6	8	5	2	4	9	1
F27	Mean	9.30*E* + 02	6.85*E* + 02	1.32*E* + 03	2.43*E* + 03	7.75*E* + 02	8.27*E* + 02	1.03*E* + 03	3.04*E* + 03	9.73*E* + 02
	Std.	2.09*E* + 02	1.14*E* + 02	2.56*E* + 02	5.22*E* + 02	1.83*E* + 02	2.09*E* + 02	2.35*E* + 02	4.72*E* + 02	1.13*E* + 02
	Rank	4	1	7	8	2	3	6	9	5
F28	Mean	9.26*E* + 03	3.77*E* + 06	1.01*E* + 06	1.47*E* + 07	1.27*E* + 04	7.34*E* + 03	4.09*E* + 05	3.98*E* + 07	2.08*E* + 03
	Std.	4.95*E* + 03	2.70*E* + 06	6.08*E* + 05	1.01*E* + 07	5.29*E* + 03	3.49*E* + 03	4.56*E* + 05	2.31*E* + 07	1.46*E* + 02
	Rank	3	7	6	8	4	2	5	9	1

**Table 4:** Statistical results of Wilcoxon signed-rank test for CEC2017.

AEO				GWO				HHO				AOA			

*P*-value	*R*+	*R*−	Win	*P* value	*R*+	*R*−	Win	*P*-value	*R*+	*R*−	Win	*P* value	*R*+	*R*−	Win
5.15*E* − 10	1326	0	+	5.15*E* – 10	1326	0	+	5.15*E* − 10	1326	0	+	5.15*E* − 10	1326	0	+
0.071907	855	471	=	5.15*E* – 10	1326	0	+	5.15*E* − 10	1326	0	+	5.15*E* − 10	1326	0	+
3.94*E* − 09	35	1291	−	5.46*E* – 10	1	1325	−	0.001534	1001	325	+	5.15*E* − 10	1326	0	+
5.15*E* − 10	1326	0	+	5.15*E* – 10	1326	0	+	5.15*E* − 10	1326	0	+	5.15*E* − 10	1326	0	+
5.43*E* − 08	1243	83	+	3.94*E* – 09	35	1291	−	5.15*E* − 10	1326	0	+	5.15*E* − 10	1326	0	+
5.15*E* − 10	0	1326	−	7.35*E* – 10	6	1320	−	5.46*E* − 10	1	1325	−	6.93*E* − 10	1321	5	+
5.15*E* − 10	1326	0	+	5.15*E* – 10	1326	0	+	5.15*E* − 10	1326	0	+	5.15*E* − 10	1326	0	+
5.15*E* − 10	0	1326	−	5.15*E* – 10	0	1326	−	5.15*E* − 10	0	1326	−	5.15*E* − 10	0	1326	−
3.96*E* − 07	1204	122	+	5.15*E* – 10	1326	0	+	1.40*E* − 09	1309	17	+	5.15*E* − 10	1326	0	+
5.15*E* − 10	1326	0	+	5.15*E* – 10	1326	0	+	5.15*E* − 10	1326	0	+	5.15*E* − 10	1326	0	+
5.15*E* − 10	1326	0	+	5.15*E* – 10	1326	0	+	5.15*E* − 10	1326	0	+	5.15*E* − 10	1326	0	+
7.35*E* − 10	1320	6	+	5.15*E* – 10	1326	0	+	5.15*E* − 10	1326	0	+	5.15*E* − 10	1326	0	+
5.15*E* − 10	1326	0	+	5.15*E* – 10	1326	0	+	5.15*E* − 10	1326	0	+	5.15*E* − 10	1326	0	+
6.52*E* − 05	237	1089	−	1.05*E* – 09	12	1314	−	0.000153	1067	259	+	1.42*E* − 08	1268	58	+
0.276894	547	779	=	2.65*E* – 09	28	1298	−	2.97*E* − 09	1296	30	+	6.93*E* − 10	1321	5	+
5.15*E* − 10	1326	0	+	5.15*E* – 10	1326	0	+	5.15*E* − 10	1326	0	+	5.15*E* − 10	1326	0	+
5.15*E* − 10	1326	0	+	5.15*E* – 10	1326	0	+	5.15*E* − 10	1326	0	+	5.15*E* − 10	1326	0	+
0.004923	363	963	−	5.53*E* – 09	41	1285	−	2.11*E* − 06	1169	157	+	9.14*E* − 09	1276	50	+
1.27*E* − 08	56	1270	−	5.15*E* – 10	0	1326	−	8.61*E* − 07	1188	138	+	5.15*E* − 10	1326	0	+
0.205722	798	528	=	5.15*E* – 10	1326	0	+	5.15*E* − 10	1326	0	+	5.15*E* − 10	1326	0	+
0.264661	782	544	=	6.15*E* – 10	3	1323	−	5.15*E* − 10	1326	0	+	5.15*E* − 10	1326	0	+
0.000331	1046	280	+	3.37*E* – 08	74	1252	−	5.15 − 10	1326	0	+	5.15*E* − 10	1326	0	+
0.006378	954	372	+	5.15*E* – 10	1326	0	+	6.93*E* − 10	1321	5	+	5.15*E* − 10	1326	0	+
0.035759	887	439	+	4.94*E* – 09	39	1287	−	1.53*E* − 07	1223	103	+	5.15*E* − 10	1326	0	+
5.15*E*−10	1326	0	+	3.03*E* – 08	1254	72	+	5.15*E* − 10	1326	0	+	5.15*E* − 10	1326	0	+
0.285261	777	549	=	5.15*E* – 10	1326	0	+	5.15*E* − 10	1326	0	+	5.15 *E* − 10	1326	0	+
0.205722	528	798	=	8.77*E* – 10	9	1317	−	9.66*E* − 09	1275	51	+	5.15*E* − 10	1326	0	+
5.15*E* − 10	1326	0	+	5.15*E* – 10	1326	0	+	5.15*E* − 10	1326	0	+	5.15*E* − 10	1326	0	+
16/6/6				16/0/12				26/0/2				27/0/1			

SMA				MRFO				PFA				BOA			

*P*-value	*R*+	*R*−	Win	*P* value	*R*+	*R*−	Win	*P* value	*R*+	*R*−	Win	*P* value	*R*+	*R*−	Win
5.15*E* − 10	1326	0	+	5.15*E*−10	1326	0	+	5.15*E*−10	1326	0	+	5.15*E*−10	1326	0	+
5.15*E* − 10	1326	0	+	0.100931	838	488	+	5.15*E*−10	1326	0	+	5.15*E*−10	1326	0	+
5.15*E* − 10	0	1326	−	3.25*E* – 07	118	1208	−	5.46*E* − 10	1	1325	−	5.15*E* − 10	1326	0	+
7.74*E* − 09	1279	47	+	8.77*E* – 10	1317	9	+	5.15*E* − 10	1326	0	+	5.15*E* − 10	1326	0	+
5.15*E* − 10	0	1326	−	0.880784	679	647	=	6.93*E* − 10	5	1321	−	5.15*E* − 10	1326	0	+
5.15*E* − 10	0	1326	−	1.18*E* – 09	14	1312	−	5.15*E* − 10	0	1326	−	5.15*E* − 10	1326	0	+
5.15*E* − 10	1326	0	+	5.15*E* – 10	1326	0	+	5.15*E* − 10	1326	0	+	5.15*E* − 10	1326	0	+
5.15*E* − 10	0	1326	−	5.15*E* – 10	0	1326	−	5.15*E* − 10	0	1326	−	8.25*E* − 08	1235	91	+
1.26*E* − 06	1180	146	+	0.006562	953	373	+	5.46*E* − 10	1325	1	+	5.15*E* − 10	1326	0	+
5.15*E* − 10	1326	0	+	5.15*E* – 10	1326	0	+	5.15*E* − 10	1326	0	+	5.15*E* − 10	1326	0	+
5.15*E* − 10	1326	0	+	6.15*E* – 10	1323	3	+	5.15*E* − 10	1326	0	+	5.15*E* − 10	1326	0	+
5.15*E* − 10	1326	0	+	5.15*E* – 10	1326	0	+	5.15*E* − 10	1326	0	+	5.15*E* − 10	1326	0	+
5.15*E* − 10	1326	0	+	5.80*E* – 10	1324	2	+	5.15*E* − 10	1326	0	+	5.15*E* − 10	1326	0	+
2.23*E* − 09	25	1301	−	4.42*E* – 09	37	1289	−	1.69*E* − 07	105	1221	−	5.15*E* − 10	1326	0	+
0.785752	692	634	=	0.004378	359	967	−	0.052342	456	870	=	5.15*E* − 10	1326	0	+
5.15*E* − 10	1326	0	+	5.15*E* – 10	1326	0	+	5.15*E* − 10	1326	0	+	5.15*E* − 10	1326	0	+
5.15*E* − 10	1326	0	+	5.46*E* – 10	1325	1	+	5.15*E* − 10	1326	0	+	5.15*E* − 10	1326	0	+
1.49*E* − 05	201	1125	−	8.49*E* – 06	188	1138	−	0.174098	518	808	=	5.15*E* − 10	1326	0	+
5.15*E* − 10	0	1326	−	1.05*E* – 09	12	1314	−	5.15*E* − 10	0	1326	−	5.15*E* − 10	0	1326	−
5.15*E* − 10	1326	0	+	1.31*E* – 05	198	1128	−	7.32*E* − 09	1280	46	+	5.15*E* − 10	1326	0	+
5.15*E* − 10	0	1326	−	3.87*E* – 05	224	1102	−	1.25*E* − 07	99	1227	−	6.93*E* − 10	1321	5	+
1.86*E* − 08	63	1263	−	0.002316	338	988	−	4.64*E* − 08	80	1246	−	5.15*E* − 10	1326	0	+
9.93*E* − 07	1185	141	+	0.004509	966	360	+	2.94*E* − 07	1210	116	+	5.15*E* − 10	1326	0	+
1.08*E* − 08	53	1273	−	0.055852	867	459	=	4.37*E* − 05	227	1099	−	8.77*E* − 10	1317	9	+
0.00044	1038	288	+	6.53*E* – 10	1322	4	+	9.87*E* − 10	1315	11	+	5.15*E* − 10	1326	0	+
5.15*E* − 10	1326	0	+	0.425599	748	578	=	3.52*E* − 09	1293	33	+	5.15*E* − 10	1326	0	+
7.43*E* − 08	89	1237	−	7.63*E* – 05	241	1085	−	0.119709	829	497	=	5.15*E* − 10	1326	0	+
5.15*E* − 10	1326	0	+	5.15*E* – 10	1326	0	+	5.15*E* − 10	1326	0	+	5.15*E* − 10	1326	0	+
16/1/11				14/3/11				16/3/9				27/0/1			

**Table 5:** Threat source settings.

Threat	Type	Position (km)	Radius (km)	Height
Threat 1	Rader	(35, 20)	13	2.8
Threat 2	Missile	(35, 52)	8	2.9
Threat 3	Artillery	(52, 72)	8	3.0
Threat 4	Missile	(63, 45)	10.7	2.9
Threat 5	Rader	(78, 78)	9	3.1
Threat 6	Artillery	(87, 45)	7	3.0

**Table 6:** Statistical results of the Friedman test.

Algorithm	Mean	B_set_	Worst	Std.	Success (%)
BOA	9.25*E* + 03	2.11*E* + 02	4.03*E* + 04	1.03*E* + 04	43.33
MRFO	6.59*E* + 03	2.20*E* + 02	3.03*E* + 04	8.51*E* + 03	56.67
SMA	2.91*E* + 03	2.17*E* + 02	2.03*E* + 04	6.41*E* + 03	83.33
GDEBOA	2.22*E* + 02	1.94*E* + 02	2.25*E* + 02	5.41*E* + 00	100.00

## Data Availability

The data used to support the findings of this study are included within the article.
